# Vascular Normalization: A New Window Opened for Cancer Therapies

**DOI:** 10.3389/fonc.2021.719836

**Published:** 2021-08-12

**Authors:** Ting Yang, Hongqi Xiao, Xiaoxia Liu, Zhihui Wang, Qingbai Zhang, Nianjin Wei, Xinggang Guo

**Affiliations:** ^1^Department of General Surgery, The Fourth Affiliated Hospital of Harbin Medical University, Harbin, China; ^2^Department of General Surgery, The Second Affiliated Hospital of Harbin Medical University, Harbin, China

**Keywords:** antiangiogenesis, tumor vessel normalization, immunotherapy, microRNA, endothelial cell metabolism, extracellular matrix

## Abstract

Preclinical and clinical antiangiogenic approaches, with multiple side effects such as resistance, have not been proved to be very successful in treating tumor blood vessels which are important targets for tumor therapy. Meanwhile, restoring aberrant tumor blood vessels, known as tumor vascular normalization, has been shown not only capable of reducing tumor invasion and metastasis but also of enhancing the effectiveness of chemotherapy, radiation therapy, and immunotherapy. In addition to the introduction of such methods of promoting tumor vascular normalization such as maintaining the balance between proangiogenic and antiangiogenic factors and targeting endothelial cell metabolism, microRNAs, and the extracellular matrix, the latest molecular mechanisms and the potential connections between them were primarily explored. In particular, the immunotherapy-induced normalization of blood vessels further promotes infiltration of immune effector cells, which in turn improves immunotherapy, thus forming an enhanced loop. Thus, immunotherapy in combination with antiangiogenic agents is recommended. Finally, we introduce the imaging technologies and serum markers, which can be used to determine the window for tumor vascular normalization.

## Introduction

Since cancer ranks the top killer of human all over the world and approximately accounts for one out of every six deaths, cancer treatment is certain to be at the epicenter of medical research (1). Angiogenesis, which provides nutrients and oxygen for tumor proliferation, is a fundamental requirement for tumor growth, showing targeting tumor angiogenesis would be a promising method to inhibit tumor growth (2).

When exposed to excessive proangiogenic signaling and hypoxia, vascular endothelial cells (ECs), called “phalanx” cells in established vessels, can quickly adapt and switch their phenotype to the so-called “tip” and “stalk,” with tip cells acting as a guide to new blood vessels and extending filopodia and stalk cells being responsible for elongating new blood vessels (3, 4). Besides, cancers can initiate some underlying angiogenesis mechanisms. For example, cancer cells can induce the formation of new vessels or use preexisting vessels (vascular co-option) to maintain growth. The former includes two new vessels splitting from one when interstitial tissue pillars insert into the lumen (intussusception) and new vessels forming from bone marrow-derived endothelial progenitor cells (postnatal vasculogenesis). Vasculogenic mimicry and mosaic vessel formation refer to the transdifferentiation of tumor cells into ECs and the incorporation of tumor cells into the blood vessel wall, respectively (5–7). Although tumor vessels are abundant, the structure and function of these vessels are abnormal, thus creating a hypoxic tumor microenvironment (TME), impeding the infiltration and function of immune cells, and promoting tumor development and metastasis (2, 7). Conventional approaches have focused primarily on inhibiting angiogenesis in tumors by cutting off their blood supply (2, 8). Several antiangiogenic drugs have been approved for the treatment of cancer. However, these antiangiogenic drugs have limited efficacy and drug resistance. Moreover, some antiangiogenic drugs reduce oxygen supply, increase the production of proangiogenic factors, and promote pathological angiogenesis, thus leading to the increased tumor metastasis and relapse in some cancers (9, 10).

**Figure 1:**
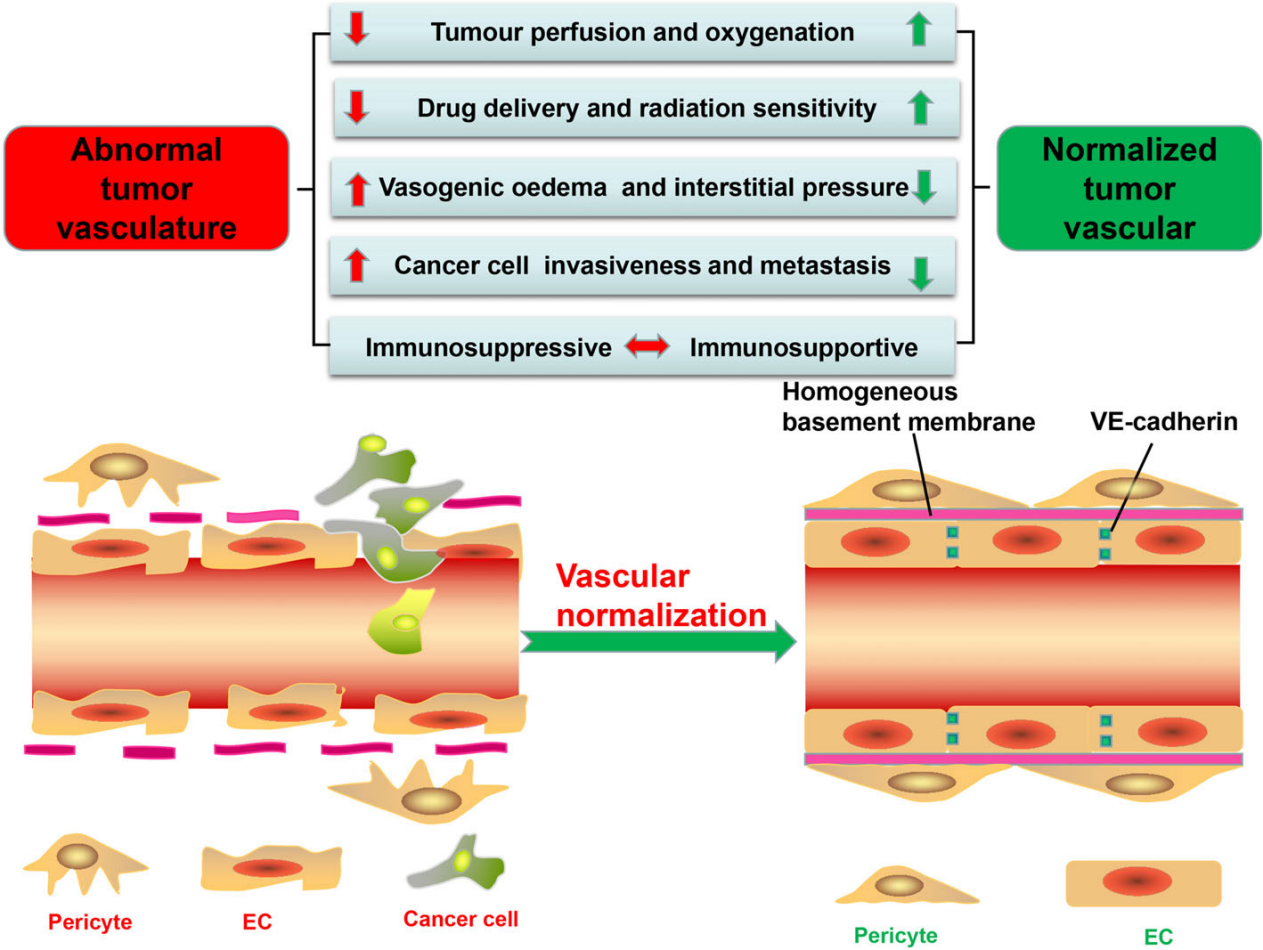
Significant differences in structure and function between tumor vessels and normal vessels. tECs have an irregular shape and lack VE-cadherin, leading to high permeability and disordered arrangement. Besides, tumor vessels have low pericyte coverage and are often separated from endothelial cells (ECs). The basement membrane of tumor vessels is also incomplete and discontinuous, further increasing vascular permeability. These structural abnormalities stimulate the formation of a hypoxic, acidic tumor microenvironment (TME); promote the proliferation and metastasis of tumor cells; and reduce the efficacy of tumor therapy. Furthermore, the abnormal TME promotes the immunosuppression. The methods of tumor vascular normalization introduced in this paper can restore the normal structure and function of blood vessels and enhance the efficacy of antitumor therapy.

A good alternative therapy for antiangiogenic explored in this study is remodeling tumor blood vessels to restore their structure and function, known as vascular normalization, which can, by restoring tumor perfusion and reducing hypoxia, not only prevent cancer cells from acquiring the aggressive phenotypes under the hypoxic microenvironment but also be conducive to other cancer therapies, such as chemotherapy, radiotherapy, and immunotherapy (11) (**Figure 1**). In this review, the methods and mechanisms of promoting tumor vascular normalization in recent years are introduced, the relationship between tumor vascular normalization and immunotherapy is discussed, and the imaging technologies and serum markers used to determine the window for tumor vascular normalization are summarized.

## Highly Abnormal Tumor Vessels

### The Abnormal Vascular Structure of the Tumor

Tumor vessels, aberrant in structure and function and heterogeneous in lumen size and wall thickness, form a chaotic network characterized by permeability and tortuousness (12–14), with perivascular cells, comprised of pericytes and vascular smooth muscle cells, not only displaying abnormal morphology but also having low coverage rate on tumor vessels compared with normal vessels, thus making the wall of the vessel thin and disrupting cellular connections and signal transduction between perivascular cells with ECs and basement membrane (15, 16) (**Figure 1**). In normal tissues, pericytes, close to ECs, communicate by intimating cell-to-cell contact and mutually secreted factors, leading to an orderly arrangement of ECs and forming an effective and organized mature blood vessel network (17). While in the tumor vessels, they usually detach from the vascular walls, resulting in decreased vascular structural stability and increased vascular permeability (7, 18–20) (**Figure 1**).

In addition, tumor endothelial cells (tECs) lining vessels have weak junctions and are disorganized (7). Responsible for the connection between vascular ECs thus maintaining the barrier integrity is VE-cadherin, which can be broken down by proteolytic enzymes (such as matrix metalloproteinases, trypsin, or elastase) and inflammatory cytokines released by tumor cells (21, 22) (**Figure 1**). For example, histamine not only directly impairs human umbilical vein ECs but also indirectly damages VE-cadherin, thereby increasing the permeability of umbilical vein (23). Besides, excessive vascular endothelial growth factor (VEGF), produced by TME, can also increase vascular permeability by regulating several GTPases such as RhoA (24), with ECs immigrating from their resident site and leaving gaps in the absence of VE-cadherin (6, 12, 13). Finally, Ang overexpressed in ECs can decrease endothelial integrity and disrupt pericyte attachment to the endothelial wall (11, 25).

Although tumor blood vessels, compared with normal blood vessels, are almost covered by incomplete and discontinuous basement membrane, confocal and electron microscopy can help discover the vascular basement membrane in tumors with such distinctive abnormalities such as unusual thickness, multiple layers, and weak junction with tECs and pericytes (6, 26), leading to the conclusion that the structure of tumor blood vessels is loose and irregular with high permeability (**Figure 1**).

### Abnormal Function of Tumor Blood Vessels

Despite a rich blood supply, the network of tumor blood vessels is chaotic and lacks the arteriole–capillary–venule hierarchical organization, leading to poor tissue perfusion (12, 15, 27) (**Figure 1**). Permeable tumor blood vessels facilitate plasma and proteins into the surrounding interstitial space, which not only enhances red blood concentration and blood viscosity but also elevates the interstitial fluid pressure in the TME, further hindering blood flow (27, 28). Moreover, the TME with high quantity of fibers and cells restricts tumor progress and induces the mechanical force which can compress or even collapse intratumoral vessels and severely retard the blood flow into and from tumors (7, 27, 29). The mechanical force also damages lymphatic networks and blocks lymphatic drainage of excessive interstitial fluid (15, 27). All these factors result in increased interstitial fluid pressure, which, in collaboration with poor perfusion, decreases oxygen and nutrient supply, leading to a hypoxic and acidic microenvironment conducive to screening out more aggressive tumor cells for survival, with their ability to metastasize and invade increased by undergoing an “epithelial to mesenchymal transition” (20, 30, 31) (**Figure 1**). However, abnormal blood vessels also disrupt the blood supply of the tumor, leaving drugs and cytotoxic T lymphocytes (CTLs) from the circulating blood unable to get into the tumors to play their full antitumor role (11, 32).

## Methods of Tumor Vascular Normalization

### Blocking Angiogenic Factors

The imbalance of signaling mediated by proangiogenic *versus* antiangiogenic molecules is one of the main mechanisms for abnormal tumor vascular (11, 33), due to the fact that, when in need of the nutrient and oxygen, tumor and stromal cells start secreting proangiogenic factors, such as the VEGF, angiopoietins, platelet-derived growth factors, and fibroblast growth factor, promoting the formation of excessive abnormal blood vessels (34, 35), indicating that targeting tumor blood vessels has become an attractive anticancer therapy approach. Antiangiogenic treatments are thought to have a good anticancer effect by inhibiting blood vessel production, thus leaving cancer cells in starvation with the supply of oxygen and nutrients blocked (8, 20, 36). However, there is a fact that antiangiogenic monotherapy is less effective in colorectal cancer, breast cancer, and nonsmall cell lung cancer, while when combined with chemotherapy drugs, anticancer effect increased significantly (37, 38), which seems to be contradictory to the traditional claim of antiangiogenesis reducing the blood perfusion and further restricting the intratumoral delivery of coadministered drugs. Besides, the hypoxic microenvironment caused by antiangiogenic therapy also renders tumor cells relatively resistant to chemoradiotherapy and even more aggressive (20, 39). By means of transiently normalizing tumor vessels with antiangiogenic therapy, the hypothesis called “vascular normalization” could explain this paradox (33).

Among all vascular angiogenic factors, VEGF, which was first identified as vascular permeability factor by Napoleone Ferrara, promotes EC proliferation and migration, sprouting *via* binding VEGF receptor–tyrosine kinase-dependent receptor on ECs (38, 40–42). The evidence, from preclinical and clinical studies, shows that blocking VEGF signaling can remodel abnormal tumor vasculature (13, 20, 43). For example, the Willett CG study showed that, after receiving a low dose of bevacizumab (5 mg/kg), patients with rectal cancer showed an increase in pericyte coverage of blood vessels and drug delivery and a decrease in permeability surface product after 12 days, while after receiving a higher dose of bevacizumab (10 mg/kg), patients showed no sign of such phenomenon, indicating that a low dose of bevacizumab not only reduced the formation of pathological blood vessels but also repaired defective blood vessels and normalize them (38, 44). This idea is also supported by bevacizumab combined with chemotherapy, with the overall and progress-free survival improved by adding a low dose, rather than a higher dose, of bevacizumab to chemotherapeutic drug in patients with metastatic colorectal cancer (37, 45, 46), indicating that an appropriate dosage of VEGF inhibitors can rebalance angiogenic signals in tumors, resulting in the formation of more mature blood vessels by actively recruiting pericytes and strengthening cell–cell connections (47, 48). Furthermore, anti-VEGF has been shown to increase platelet-derived growth factor receptor-β signaling and promote pericyte recruitment and decrease permeability (49). Regarding the phenomenon that anti-VEGF therapy increased pericyte coverage while causing vessel regression, Hanahan and Bergers postulated that this is an unconventional mode of resistance that the high coverage of pericytes can help tECs to survive and function, so that tumors can still grow slowly in antiangiogenesis treatments (50).

Despite that anti-VEGF therapy has the potential to restore abnormal tumor vascular normality, there are still some challenges, of which the major one is the time range and dose of anti-VEGF administration to achieve vascular normalization (15, 20), known as the “normalization window,” which was observed to last for only several days after treatment began, providing a narrow time window for the delivery of drugs (13, 20, 48, 51). However, tumors begin to grow again after adaptive resistance, preexisting nonresponsiveness, and antiangiogenic therapy drug withdrawal, leaving many patients failing to produce enduring clinical responses (48, 50, 52, 53). The transient effect of tumor vascular normalization might be associated either with excessively high and continuous administration of antiangiogenic drugs or the occurrence of drug resistance by activating other proangiogenic factors, such as angiopoietins, basic fibroblast growth factor, and transforming growth factor-b (TGF-β) (2, 13, 50, 54). High doses of antiangiogenic drugs degenerate blood vessels, increase tumor hypoxia, and stimulate invasion, infiltration, and metastasis of cancer cells (20, 55, 56), and the heterogeneity of the response of different tumors to VEGF inhibitors further increases the difficulty of achieving the normalization window. Moreover, the discovery of VEGF receptors on nonendothelial cells, specifically VEGFR2, further increases the complexity of VEGF research. MET, the receptor of hepatocyte growth factor, is correlated with increased tumor invasion and poor survival in glioblastoma multiforme. The study found that VEGF blockade restored and increased MET activity in a hypoxia-independent manner while enhancing mesenchymal characteristics in mouse models of glioblastoma multiforme (57, 58). It has been revealed in clinical practices that antiangiogenic drugs have caused some serious side effects, such as hypertension, venous thromboembolism, spontaneous internal bleeding, gastrointestinal perforation, cardiac toxicity, and endocrine dysfunction (54, 59, 60).

Receptor tyrosine kinase inhibitor, another class of antiangiogenic drugs such as sorafenib, was approved by the U.S. Food and Drug Administration to inhibit VEGFR and platelet-derived growth factor receptor-β, leading to the enhanced VE-cadherin junctions between ECs and pericyte recruitment (61–63). The mechanism of VEGFR2 blockade leads to vascular normalization *via* angiopoietin-1 (Ang1)/Tie2 signaling (64); thus, recent studies aimed at its role involved in the regulation of vascular normalization (13, 55, 65–67). Ang1, expressed primarily on perivascular cells, acts as a stabilizer for the tumor vasculature through Tie2 activation (68–70). However, Ang2, predominantly produced by ECs in hypoxic TME, not only competes with Ang1 for the binding of Tie2 and deactivates it, but also promotes tumor vessels sprouting in the presence of angiogenic growth factors including VEGF (71–74). Ang2 blockade promotes vessel normalization *via* reducing pericyte dropout and strengthening endothelial junctions of the tumor vasculature (71, 75, 76). Vascular endothelial protein tyrosine phosphatase (VE-PTP) inactivates Tie2, so blocking VE-PTP can promote tumor vascular maturation and normalization (77). Since single blockade of Ang2 may be insufficient to induce vessel normalization, dual blockade of Ang2 and VEGF can reprogram tumor-associated macrophages and promote the effector function of T cells, which can enhance the efficacy of tumor vascular normalization and has been shown to increase the efficacy of immune checkpoint blockade (ICB) (78–80). Dual blockade of Ang2 and VEGF has been shown to prolong survival in mouse models of glioblastoma, melanoma, pancreatic neuroendocrine tumors (PNETs), and metastatic or early stage (resected) breast cancer, except for rectal cancer (11, 81–83). These findings prove that the effect of dual blockade of Ang2 and VEGF is closely related to tumor type, stage, and TME. For the dosing and scheduling of VEGF and ANG2 blockers, further studies are needed (11). Besides, compared with Ang2 blockade alone, the vascular stabilization and perfusion were simultaneously increased by the combined targeting of Tie2 and Ang2 (84). However, research studies have shown that blocking both Ang1 and Ang2 might not be of benefit to patients (85–87). The possible reason is that blocking Ang1 might compromise the benefits of vascular normalization conferred by the blockade of the antagonistic TIE2 ligand ANG2 (11). Notably, both single and double blocking strategies are still posing the risks of increasing tumor hypoxia and promoting TME degradation (20).

### Inducing Angiostatic Factors

As mentioned above, inhibition of proangiogenic signals can promote the normalization of tumor vessels, which can also be achieved by increasing angiostatic factors such as tumor necrosis factor α (TNFα), thrombospondin-1 (TSP-1), and endostatin. Direct injection of low-dose TNF-α into tumors stabilizes tumor vascular network, improves vascular perfusion, and substantially enhances antitumor vaccination or adoptive T-cell therapy (88, 89). The tumor vasculature could be normalized and the effector tumor infiltrating lymphocyte (TIL) infiltration could be improved by a member of the tumor necrosis factor superfamily, LIGHT, also known as herpesvirus entry mediator ligand (90–92), which has been shown, when bound to a tumor vascular targeting peptide (VTP), to be able to repair abnormal tumor vasculature by increasing the expression of intercellular adhesion molecule-1 (ICAM-1), vascular cell adhesion protein-1 (VCAM-1), smooth muscle actin, caldesmon, and calponin (90, 93, 94). All of these lymphotoxin β receptor (LTβR)-dependent pericyte contractile markers can strengthen cell-to-cell connections and reduce leakage of blood vessels. LIGHT also activates the intratumoral macrophages to secrete TGF-β, which increases the synthesis of adhesion molecules in a Rho-kinase-dependent manner and modulates pericyte differentiation. LIGHT-driven tumor vascular normalization has been shown to be achieved by improving the pericyte coverage rate of blood vessels and enhancing the effect of cancer therapy (3, 90, 95, 96). Furthermore, LIGHT–LTβR signaling induces the formation of high endothelial venules, which are the primary sites where leukocyte, especially TIL, extravasates from blood vessels into target tissues (94, 97). Although some research studies have shown that LIGHT could be used as a monotherapy, the most effective way is to combine it with vaccinations or checkpoint inhibitors to promote vascular normalization and enhance cancer treatment (94, 98, 99). However, in clinical practices, a LIGHT-based therapy should take into consideration the interfering effect of the tumor necrosis factor receptor superfamily member 6b, known as decoy receptor 3 (DcR3), because LIGHT signaling could be attenuated by DcR3. Owing to the fact that DcR3 expression was detected in neither mice nor rats, further explorations need to be conducted for the purpose of confirming whether LIGHT plays a role in promoting vascular normalization in the presence of DcR3 (100–102).

TSP-1, produced by stromal cells and cancer cells, can induce tumor vascular normalization. ABT-510, one of the mimics of TSP-1, is able to promote the normalization of vascular structure and function without reducing vascular density (103). In addition to normalize tumor blood vessels by directly increasing TSP-1 levels, metronomic chemotherapy, defined as frequent administration at lower doses, also reprograms the tumor immunosuppressive microenvironment into immune stimulation, thus kills cancer cells and reduces the pressure on tumor blood vessels, resulting in the promotion of the tumor blood vessel normalization (20, 104–107).

Besides, studies have shown that TSP-1 related to the normalization of tumor vessels is induced by moderate aerobic exercise, which alone promoted melanoma growth but had no effect on pancreatic ductal adenocarcinoma growth, possibly owing to the specific effect of exercise on tumor growth. However, on the basis of further research, antitumor efficacy in both types of tumors was increased by combining exercise with chemotherapy, as a result of exercise-induced shear stress activating calcineurin–NFAT–TSP-1 signaling in ECs and promoting tumor vascular normalization in mouse models (108–111). Moreover, the level of TSP-1 had been found in recent studies to be usually elevated around normal vessels compared with abnormal vessels, suggesting that TSP-1 may be a marker of vascular normalization (112).

EC proliferation and migration, as well as the expression of VEGF, could be inhibited by an angiogenesis inhibitor, recombinant human endostatin (endostar), which was shown, with recent evidence, to possibly restore vascular homeostasis and induce vascular normalization in some cancer (112, 113). However, still unclear is the molecular mechanism, which, in studies, was shown that the Src signaling pathway and matrix metalloproteinase (MMP) might be the mechanism of its action (114, 115). As mentioned above, inhibiting proangiogenic signaling or enhancing antiangiogenic signaling can prune some abnormal vessels and fortify the remaining, resulting in a normalized vasculature (20).

### Targeting EC Metabolism

Endothelial cell metabolism has become a new way to promote tumor vessel normalization. Although located next to the bloodstream and has the capacity to obtain oxygen easily, ECs surprisingly produce up to 85% of their total cellular adenosine-triphosphate (ATP) content *via* aerobic glycolysis—a phenomenon known as the Warburg effect (116–118). Angiogenesis requires abundant ATP and biomass to proliferate and migrate ECs, especially the active tip and stalk cells (119). However, ECs select glycolysis to produce ATP, which is less efficient than oxidative phosphorylation for the following reasons (116, 120): first, regulation of glycolytic flux occurs faster than oxidative phosphorylation, which ensures an immediate supply of energy when the ECs proliferate and migrate (121); second, ATP, only produced by glycolysis rather than oxidative phosphorylation, provides energy for EC migration, and the intermediates of glycolysis provide the precursors of biomass synthesis for EC proliferation (122–124); third, *via* aerobic glycolysis, metabolism reduces the release of reactive oxygen species and protects ECs from oxidative stress (116); and fourth, high glycolysis reduces oxygen consumption, thus ensuring adequate oxygen supply to surrounding cells (125, 126).

**Figure 2:**
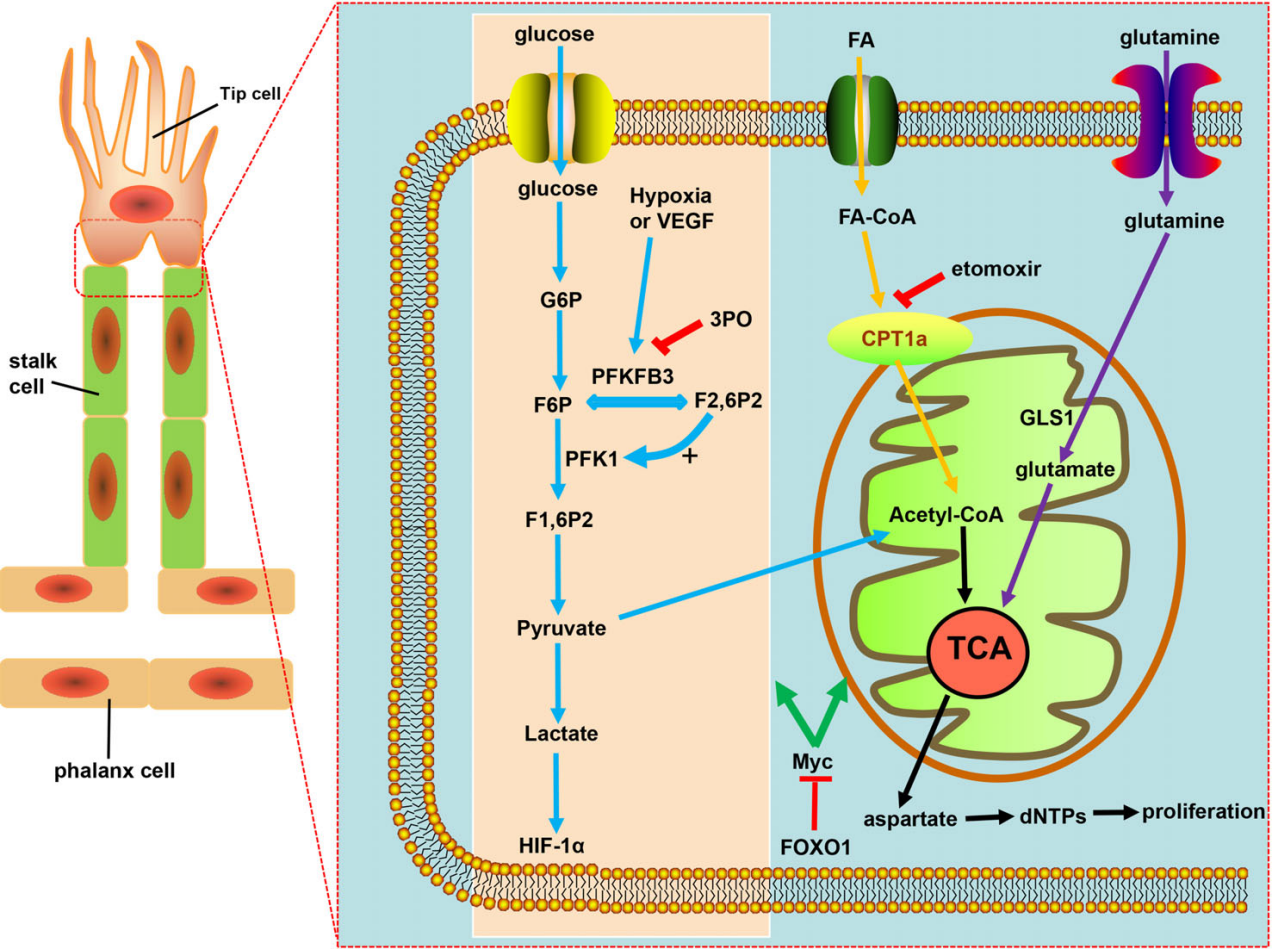
Targeting EC metabolism promotes tumor vascular normalization. Glycolysis fuels the proliferating ECs. PFKFB3, overexpressed under hypoxia and vascular endothelial growth factor (VEGF) stimulation, is a key regulator of the glycolytic activity in ECs. F2,6P2, produced from F6P by PFKFB3, is the main allosteric activator of PFK1, which further increases glycolysis to produce ATP necessary for angiogenesis. PFKB3 inhibitor 3PO reduces pathological angiogenesis. CPT1a-driven FAO and glutamine metabolism both regulate angiogenesis by replenishing the TCA cycle and producing aspartate for nucleotides and DNA synthesis. CPT1a inhibition with etomoxir can promote tumor vessel normalization. FOXO1 keeps EC quiescence and vascular homeostasis *via* c-Myc regulation, ultimately resulting in decreased glycolysis and impaired mitochondrial function.

During the process of angiogenesis, stimulation by VEGF leads to increased glycolysis in ECs and displays increased expression of 6-phosphofructo-2-kinase/fructose-2,6-biphosphatase 3 (PFKFB3) (116, 127). Hyperglycolysis accumulates excess lactic acid, which is utilized by ECs and activates hypoxia-inducible factor 1α (HIF-1α), thus leading to pathological angiogenesis (116). PFKFB3, an indirect component of the glycolytic pathway, can synthesize fructose-2,6-bisphosphate (F2,6BP), thus allosterically activating phosphofructokinase 1 (PFK1), a rate-limiting enzyme of glycolysis (116, 128, 129) (**Figure 2**). Compared with normal proliferating ECs, tECs show an even higher reliance on PFKFB3, of which the additional expression leads to a more immature and dysfunctional vasculature, *via* activating tip cell behavior and enhancing stalk cell proliferation (119, 130).

The glycolysis of ECs was partially reduced by the inhibition of glycolytic activator, PFKFB3, either with low-dose pharmacological PFKFB3 blockade with the small molecule 3-(3-pyridinyl)-1-(4-pyridinyl)-2-propen-1-one (3PO) or by endothelial PFKFB3 haplodeficiency, with the pathological angiogenesis blocked without causing excessive vascular pruning (7, 131) (**Figure 2**). Moreover, PFKFB3 inhibition also downregulates the expression of adhesion molecules such as VCAM-1 and ICAM-1 by inhibiting NF-kB activity, thereby weakening the adhesive interaction between tumor cell and ECs, leading to the reduced cancer cell invasion and metastasis (119, 129, 132). Mechanistically, PFKFB3 blockade reduces the endocytosis of VE-cadherin and increases the expression of N-cadherin expression in pericytes, leaving the adhesion to ECs enhanced to keep more quiescent, thus promoting the barrier function of tumor blood vessels (132, 133). However, deletion of both PFKFB3 alleles in ECs or the excess of PFKFB3 inhibitor 3PO inhibited tumor growth due to reduced tumor perfusion and induced systemic toxicity, which proved that the inhibition degree of PFKFB3 determines its effect on the tumor vascular system (119, 134, 135).

Crucial to angiogenesis is, apart from the glycolytic pathway, fatty acid oxidation, on which stalk cells depend to promote vessel sprout elongation through deoxynucleotide triphosphate (dNTP) synthesis. Fatty acids (FAs) were imported into the mitochondria by carnitine palmitoyltransferase 1a (CPT1a), a rate-controlling enzyme of FA breakdown, then underwent β-oxidation to produce acetyl-CoA, which, in conjunction with anaplerotic substrate, sustains the tricarboxylic acid cycle (TCA) for dNTPs and DNA synthesis in proliferating ECs (119), resulting in the decrease in dNTP synthesis and blockade of pathological angiogenesis caused by pharmacological blockade of CPT1a with etomoxir as well as endothelial cell-selective genetic deletion of CPT1a (116, 136, 137) (**Figure 2**). ECs can harness glutamine metabolism to provide energy, and drugs that inhibit glutaminase (GLS1) can normalize tumor blood vessels (7, 138, 139) (**Figure 2**). Forkhead box O 1 (FOXO1), a member of the FOX transcription factors family, was found in recent investigations to keep EC quiescence by impairing mitochondrial function and reducing glycolysis, playing its role by inhibiting Myc protooncogene protein (c-Myc)—a key transcriptional factor in growth and anabolic metabolism (140–142), thus, due to the importance of FOXO1 in angiogenesis and homeostasis of the vessel, raising the question: whether FOXO1 is a good target of restoring tumor vessel normalization (**Figure 2**).

### MicroRNAs

Encoded by endogenous genes with approximately 22 nucleotides, microRNAs are single-stranded noncoding RNAs that can regulate mRNA expression in the development and progression of tumors, of which some can restore the integrity of tumor vascular structure by regulating EC functions during angiogenesis (143–146). Considered to play a major role in controlling angiogenesis and vascular structural integrity, miR-126 is widely expressed in venous and arterial ECs and promotes tumor angiogenesis and maintains tumor vascular normalization by regulating downstream growth factors such as VEGF, basic fibroblast growth factor, and epidermal growth factor (147). For example, a single dose of miR-126 injected in a urine ischemic hindlimb model renders a significant reduction in capillary vessel density (148–150). MiR-15b and miR-16 in cells lead to decreased VEGFA expression affecting the blood vessel formation during tumor growth (151, 152). MiR-20b acts as a negative regulator of VEGF in breast cancer cells and nasopharyngeal carcinoma epithelioid cells under hypoxic conditions. The mechanism came into play as miR-20b inhibited the nuclear aggregation of HIF-1α and signal transducer and activation of transcription 3 (STAT3) (153, 154). The increase of HIF-1α and activated STAT3 has been shown to be effective in promoting tumor pathologic angiogenesis, while downregulation of HIF-1α and STAT3 did the opposite (155–157). MiR-100 decreases EC proliferation, migration, and invasion through the mammalian target of rapamycin (mTOR)/HIF‐1α/VEGF pathway in mesenchymal stem cells (158). MiR-107 regulates tumor suppressor gene by *p53* in human colon cancer specimens. Yamakuchi et al. found that the overexpression of miR-107 in tumor cells reduced VEGF expression and inhibited tumor angiogenesis in HCT116 cells, and miR-107 could downregulate HIF-1β expression in human colon cancer specimens, leading to the results that miR-107 can mediate *p53* regulation of hypoxic signaling and tumor angiogenesis (159). MiR-192 inhibits tumor angiogenesis, resulting in tumor regression and growth inhibition (160). MiR-221/222 can reduce EC migration and proliferation *via* targeting endothelial nitric oxide synthase (eNOS) and *c-kit* (161, 162), and miR-874 has been shown to be able to inhibit the tumor angiogenesis of gastric cancer cells *in vitro* and *in vivo* by targeting STAT3, and downregulation of miR-874 contributes to tumor angiogenesis (163). On the basis of the above analysis, it has a good prospect to promote vascular normalization through regulation of microRNAs (miRNAs). In **Table 1**, some miRNAs listed are shown to have great potential in promoting tumor vascular normalization.

**Table 1:** Some miRNAs associated with vessel normalization.

MicroRNA	Targets	Roles	References
miR-15b/miR-16	VEGFA	Induce cell apoptosis	(153)
miR-20b	HiF-1α, STAT3	Downregulating VEGF and suppressing angiogenesis	(154)
miR-100	mTOR	Inhibits vascular cell sprouting and proliferation	(158)
miR-107	HiF-1α	Suppressing angiogenesis	(159)
miR-126	VEGFA	Tumorigenicity and angiogenesis	(147, 149)
miR-192	EGR1, HOXB9	Globally downregulates angiogenic pathway	(160)
miR-221/miR-222	c-kit, eNOS	Inhibit EC migration and proliferation	(161)
miR-874	STAT3	Tumor growth and angiogenesis	(163)

EGR1, early growth response protein 1; HOXB9, homeobox (HOX) B9; eNOS, endothelial nitric oxide synthase.

### Targeting the Extracellular Matrix

Vascular normalization was also promoted by the normalization of the extracellular matrix (ECM) which contains a large number of fibers, including collagens, elastin, proteoglycans, glycoprotein, and fibronectin in the TME (164–166), owing to the fact that the dense ECM and hyperplasia of cancer cells create mechanical forces that squeeze the blood vessels, thus destroying the integrity of blood vessels and impairing perfusion (29, 164). The mechanical therapy, which reduced perivascular pressure by targeting ECM fibers or stromal cells, was proposed for the purpose of restoring vascular normalization, since degradation or depletion of ECM such as cancer-associated fibroblasts (CAFs) and hyaluronan had been shown to increase tumor perfusion and chemotherapy efficacy in preclinical studies (167–169). Desmoplasia was promoted by angiotensin II (Ang II) bond to Ang II type 1 receptor (AT1R) *via* activating TGF-β and upregulating the expression level of connective tissue growth factor in CAFs. These mechanisms regulate the generation of ECM. The angiotensin system inhibitor and direct TGF-β inhibition not only inhibit CAF activation and ECM production but also inhibit angiogenesis and enhance the effect of immunotherapy on some cancers (167, 170). As shown in studies, inhibiting CXCL12/CXCR4 could improve the distribution of chemotherapy agents by effectively relieving stress in tumors, while inducing stromal cell depletion does not relieve tumor solid stress (171, 172). In conclusion, matrix normalization is a good strategy to restore tumor vascular structure and potentiate chemotherapy (173–175).

### Other Methods

The vascular normalization can also be promoted by, in addition to the above methods for tumor vascular normalization, targeting other molecules in the TME. For example, transient receptor potential vanilloid 4 (TRPV4), a mechanosensitive ion channel, is a mechanosensor of shear stress and cyclic strain commonly expressed in vascular ECs, of which, compared with normal ECs, the expression and function were decreased in tECs (176–182). Regarding TRPV4 knockout mice, the vascular density increased, the pericyte coverage decreased, and the tumor growth increased. On the contrary, overexpression or pharmacological activation of TRPV4 can normalize vasculature and increase drug delivery (178, 183–186). Importantly, TRPV4 activator inhibited tumor growth in mice when used in combination with the chemotherapeutic drug cisplatin (183, 184). The mechanism of TRPV4 inducing tumor vascular normalization might be through regulation of Rho/Rho kinase *via* modulating integrin activation, with TRPV4 overexpression significantly inhibiting Rho/Rho kinase activity, which possibly accounts for the recovery of mechanical sensitivity and the normalization of angiogenesis in tECs (183, 184, 187–190). It was found in further experiments that the deletion of TRPV4 significantly increased the basal Rho and promoted tEC proliferation and migration, indicating that TRPV4 can be used as a novel therapeutic target to promote tumor vascular normalization by regulating the Rho/Rho kinase pathway (185, 191).

With G-protein signaling 5 (Rgs5) mediating abnormal tumor angiogenesis and targeting Rgs5 promoting tumor vessel normalization, RGS5-deficient mice blood vessels showed increased pericyte coverage and reduced vessel permeability, which is conducive to reducing tumor hypoxia and promoting blood perfusion to the tumor (31, 192). Able to upregulate the expression of VEGFR2 and promote endothelial sprouting is a member of the sex-determining region Y-box (SOX) transcription factor family, transcription factor Sox17, of which deletions in tECs inhibited pathological angiogenesis and promoted tumor vascular normalization. Research found that Sox17 mutant mice improved drug delivery and inhibited tumor metastasis, suggesting that long-term tumor vascular normalization is conducive to inhibiting the malignant progression (193, 194).

Oxygen levels can be sensed by an oxygen sensor, endothelial prolyl-hydroxylase 2 (PHD2), which regulates angiogenesis by targeting HIF (195). *Via* tightening the endothelial layer, the tumor vascular normalization was promoted by mice with haploid-deficient PHD2 in the ECs that were able to restore tumor vascular abnormalities by reducing CAF activation, matrix production, and contraction by CAFs (196, 197). As CAF-induced matrix deposition was a migration scaffold for cancer cell dissemination, PHD inhibition reduced lung metastasis. Moreover, PHD protein expression in lung metastases inhibited T-cell effector function and increased the recruitment of Treg cells, so its inhibition decreased Treg cell recruitment and enhanced the immune response (198, 199). C-Src, a member of the Src family kinases (SFKs), is a nonreceptor tyrosine kinase located in cells and is well known for its role in tumorigenesis. Recent findings indicated that the Src inhibitor dasatinib could maintain the integrity of ECs, thus showing the potential to restore tumor vascular normalization (200, 201). Lysophosphatidic acid receptor 4 (LPA4) could tighten endothelial cell–cell contact *via* promoting cortical actin fiber formation of ECs and stabilizing VE-cadherin, which strengthened the connections between ECs and reduced leakage of blood vessels, and when used in combination with chemotherapy drugs, LPA4 could enhance the antitumor effect (202, 203). A neural adhesion protein with the effect of promoting angiogenesis is, expressed in tumor ECs, endothelial glycoprotein L1 (L1CAM), of which the blockade with anti-L1CAM antibodies or lacking L1CAM resulted in reasonable distribution, complete structure, and normal function of tumor vessels (204). R-Ras is a GTPase that maintains EC survival and promotes vascular maturation through activation or overexpression of R-Ras in vascular smooth muscle cells or EC cells (205). Mediated by proangiogenic factors in tumors is the upregulation of MMPs, which damages vascular basement membrane making the vessel dysfunctional, indicating that inhibiting MMPs or targeting integrinα6 can block the action of MMPs and promote vascular basement membrane integrity (183, 206, 207). On the basis of studies, the antimalarial drug chloroquine had also shown promising results in normalizing tumor blood vessels in that chloroquine increased intratumoral oxygen, improved the efficacy of chemotherapy, and inhibited cancer cell metastasis, which relied mainly on an autophagy-independent, NOTCH1-reliant mechanism to promote tumor vessel normalization (208–210). Another chemotherapy drug with antiangiogenic effects, eribulin, could increase EC–pericyte interactions and form fortified vessels by modulating the expression of angiogenesis molecules (211, 212).

Taken together, new targets for promoting tumor vascular normalization might be provided by targeting the stroma, cancer cells, and other cells in the tumor environment.

## The Relationship Between Tumor Blood Vessels and Immunity

### A Vicious Circle Between Abnormal Tumor Blood Vessels and Immunosuppressive Microenvironment

**Figure 3:**
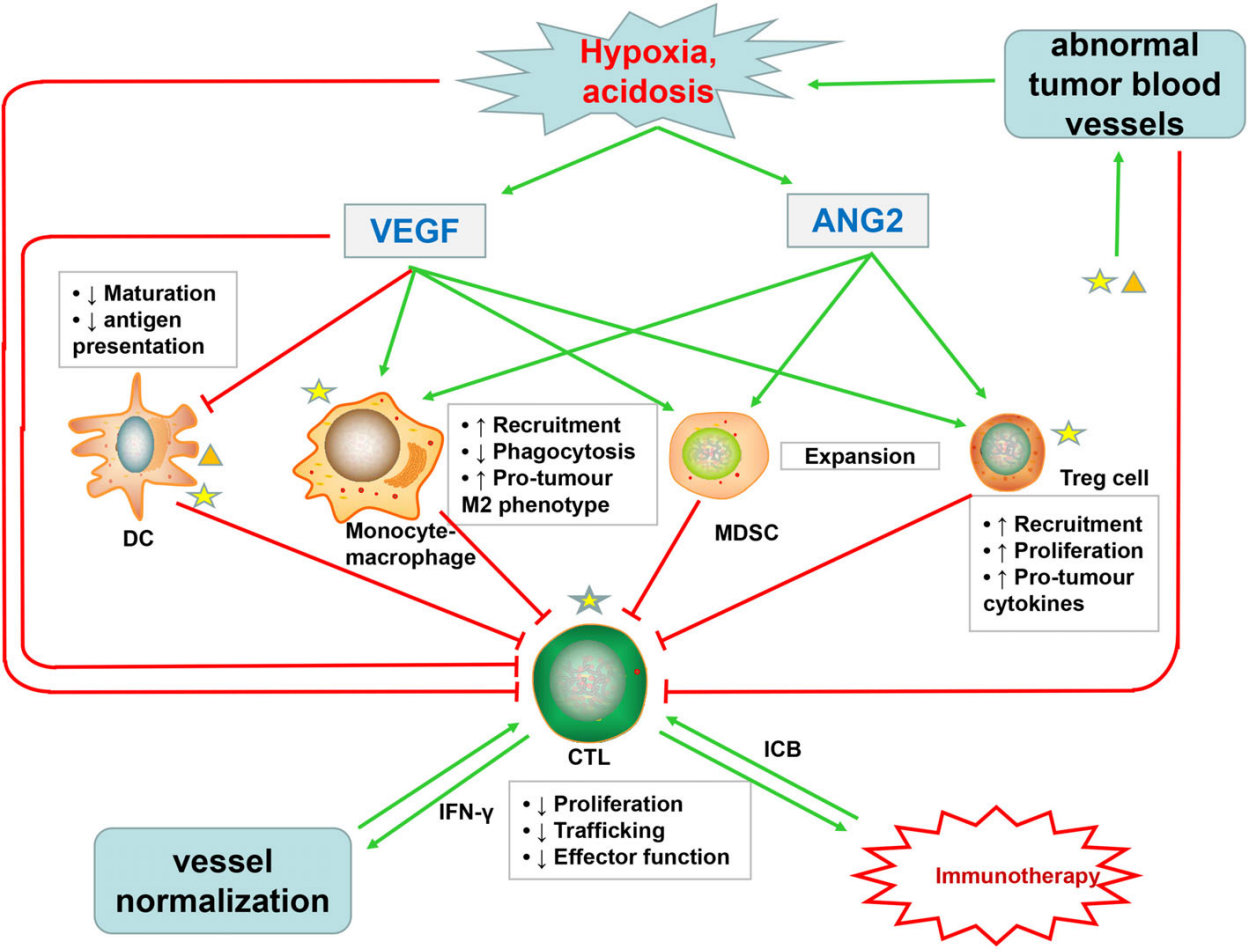
Abnormal tumor blood vessels not only inhibit the infiltration of cytotoxic T lymphocytes (CTLs) but also promote the formation of hypoxic, acidic TME, which affects the function of CTLs and increases the accumulation of VEGF and Ang2. VEGF, in addition to directly impairing the function of CTLs, also inhibits the maturation and antigen presentation of DC, which are necessary for priming of CTLs. Ang2 restricts anticancer activity by modulating the function of monocytes. Both VEGF and Ang2 promote the recruitment of immunosuppressive cells such as Treg cells, MDSCs, and TAMs. These immunosuppressive cells suppress the activity of CTLs, which promote tumor vascular normalization through secreting IFNγ. Immune cells, especially immunosuppressive cells, can produce excessive VEGF and Ang2, further promoting pathological angiogenesis. Immunotherapy with ICB can stimulate CTLs to produce IFNγ, further inducing tumor vascular normalization. The normal tumor blood vessels can promote the infiltration of CTLs and enhance tumor immunotherapy, thus forming a virtuous cycle.

Normal structure and function of blood vessels allow immune cells to enter tissues and destroy cancer cells, while the disorganized tumor vessels seem to form a selective immune cell barrier to limit the extravasation of leukocytes—especially cytotoxic T lymphocytes (CTLs)—into blood vessels (12, 20, 51, 213). Particularly, the process of immune cells adhering to the ECs and then transmigrating across the vessel wall is also blocked (214). For example, integrin ligands ICAM-1 and VCAM-1 were both expressed on ECs and immune cells, thus reducing the transport of immune effector cells and infiltration into the tumor core (215, 216). tECs, expressing the immune checkpoint protein-programmed cell death 1 ligand 1 (PD-L1), can bind to PD-1 expressed on T cells and thus inhibit its anticancer activity. The hypoxic TME leads to excessive lactic acid accumulation, thus impairing cytotoxic T-cell function *via* interfering with the production of interferon-γ (IFNγ) triggered by the T-cell receptor (TCR) (217–219) (**Figure 3**).

The abnormal tumor vasculature not only directly affects the infiltration of immune effector cells but also indirectly promotes TME-mediated immune suppression through synthesis of proangiogenic factors, such as VEGF and Ang2 (20, 220). Excessive VEGF in TME promotes immunosuppression through at least four mechanisms: first, VEGF inhibits CTL trafficking and effector function by regulating the inhibitory checkpoints of T cells (221); second, VEGF inhibits dendritic cell (DC) maturation and antigen presentation, thus further hampering T-cell activation (222); third, VEGF promotes the recruitment and proliferation of immunosuppressive cells, such as Treg cells, myeloid-derived suppressor cells, and protumor M2-like tumor-associated macrophages (TAMs) (19, 223, 224); and fourth, as described above, VEGF promotes aberrant vasculature and causes hypoxia to locally and systemically foster immunosuppression (19, 33, 225) (**Figure 3**). In addition to VEGF, activated Ang2 signaling also plays a significant role in promoting tumor immunosuppression: for one thing, Ang2 facilitates the recruitment of MDSCs (myeloid-derived suppressor cells) (226), Treg cells (227), and TIE2-expressing monocytes *via* upregulating adhesion molecules between leukocytes and ECs (228); for another, Ang2 limits the anticancer activity of monocytes by inhibiting the secretion of TNF. These proangiogenic factors might cooperate to induce tumor immunosuppression (**Figure 3**). Moreover, an optimum level of VEGF and Ang2 blockade not only promotes tumor vascular normalization but also enhances the anticancer effect of immune cells (82, 83).

Furthermore, increased activation and recruitment of immunosuppressive cells can in turn induce more abnormal angiogenesis, which form a vicious circle of disrupted immune activation (229). MDSCs can not only secrete VEGF but also induce MMP9 to act on the ECM, of which both can enhance the proliferation and migration of ECs, thus promoting angiogenesis (230). As shown in studies, angiogenesis will be promoted when VEGFR1 and VEGFR2 expressed on DC cells bind to the VEGF-A, and TAMs and monocytes can also synthesize and secrete VEGF (231, 232) (**Figure 3**). Interestingly, recent studies have shown that immunosuppression, in turn, leads to resistance to antiangiogenic treatments (233–235).

### A Virtuous Circle Between Immunotherapy and Vascular Normalization

Immunotherapy has proven effective in treating cancers and has become the standard for many cancers (236, 237), while recent findings indicated that stimulation of immune cells, especially T cells, plays an important role in the process of vascular normalization in transplant mammary tumors (238–240). Studies have shown that whether in CD4^+^ T-cell-deficient mice or CD8^+^ T-cell-deficient mice, the blood vessels were structurally and functionally abnormal, with the CD4^+^ T-cell-deficient mice particularly decreasing pericyte coverage and increasing permeability of the blood vessels, whereas manual injection of CD4^+^ T cells into tumor-bearing mice increased pericyte coverage of the blood vessels and reduced hypoxia, indicating the occurrence of tumor vascular normalization (238, 240). Through activating of immune checkpoints, cancer cells are able to block the initiation and activation of T cells. T cells, which were thought to be primarily affected by ICB with anti-PD1 and anti-cytotoxic T-lymphocyte-associated antigen 4 (CTLA4) antibodies, can promote abnormal tumor vessel normalization (241–243). The main molecular mechanism of this process is mediated by IFNγ (244), which not only inhibited tumor growth, but also acted on ECs to downregulate the expression of delta-like protein 4, thus inhibiting Notch signaling pathway, which is a key pathway of angiogenesis (245). Besides, IFNγ stimulation significantly reduced VEGF secretion in tumor-associated fibroblasts, leaving the angiogenesis further inhibited (246). Moreover, Tian et al. found a positive correlation between IFNγ secreted by Th1 cells and vascular normalization in various mouse models with vessel normalization or T-lymphocyte deficiencies (238) (**Figure 3**). ICB has been proved to be effective at improving overall survival in many cancers. However, in phase III clinical trials, ICB failed to improve overall survival in glioblastoma multiforme due in part to the immunosuppressive TME. Immune suppression was mediated in part by microglia, bone-marrow-derived myeloid cells, and granulocytes. These cells also enhanced tumor growth and resistance toward antiangiogenic therapy by expression of alternative proangiogenic factors such as CXCL2, IL8, and CD13 (247–251). In addition to T cells, the activation of eosinophils can also normalize tumor blood vessels, but the exact mechanism is unclear, which may be that the TAMs were polarized into an M1-like phenotype through eosinophil-derived IFNγ and TNF signaling, leading to a reduced low VEGF production (252). However, it was shown in a study on promoting IFNγ expression in murine fibrosarcoma and adenocarcinoma cells that that IFNγ directly inhibited angiogenesis and cut off blood flow to bring about intratumoral ischemia, by binding to IFNγ receptors on tECs. It is markedly different from the evidence that IFNγ promotes vascular normalization, which might be because the IFNγ expressed in tumor cells produced a higher and more persistent systemic concentration than the transient IFNγ elevation that can be stimulated by ICB (11, 89, 238).

Immunotherapy has the potential to promote vascular normalization, which in turn further promotes the improvement in immunotherapy effectiveness, thus forming a positive feedback loop. Restoring blood vessel normalization reduces interstitial fluid pressure and improves tumor perfusion, a process that not only increases the infiltration of immune cells within the tumor but also increases the supply of oxygen and nutrients. Adequate oxygen and nutrients can improve the overall anticancer immunotherapeutic response, as high oxygen can enhance the function of cytotoxic T cells (31, 225, 253, 254).

### The Combination of Immunotherapy and Conventional Antiangiotherapy

In order to expand the advantages of immunotherapy, the combination of immunotherapy and antiangiotherapy was investigated to improve the effect of promoting vascular normalization. Since the appropriate dose of antiangiogenic drugs can induce vascular normalization and improve the delivery of therapeutic agents to tumors, the dose of ICB can be reduced, which was sometimes known to cause severe immune-related adverse events. Recently, statistics showed that anti-PD1/PDL1 combination trials has continued to increase over the past decade, especially the combination of anti-PD1/PDL1 with VEGF/VEGFR-targeted therapies has become the top combination treatment modality (11, 255, 256). Some preclinical studies demonstrated that the combination treatment of ICB and antiangiotherapy was significantly more effective than monotherapy (257), which is supported by the instance of the findings of Lieu et al. that the combination of bevacizumab and atezolizumab could normalize the TME and inhibit tumor growth in a Cloudman melanoma mouse transplant tumor model. Zhao et al. have proven that low-dose apatinib combined with anti-PD-L1 antibody could inhibit tumor growth and prolong the survival time of a syngeneic lung cancer mouse model (258). The findings of Allen et al. suggested that the combination treatment of anti-PD-L1 therapy and antiangiogenic therapy could improve clinical anticancer efficacy by creating positive feedback loops as well (254, 259). The results of the phase I clinical trials showed that bevacizumab combined with ipilimumab (anti-CTLA4) can improve vessel morphology and increase infiltration of DCs and cytotoxic T cells in melanoma tumors (260–262). Moreover, dual VEGF–Ang2 blockade has been shown to upregulate the expression of adhesion molecules during the window of vascular normalization, thereby facilitating the accumulation of anticancer T cells within multiple types of tumors in mice. Further studies revealed that dual VEGF–Ang2 blockade leads to the upregulation of PD-L1 on ECs and tumor cells, which may be a possible mechanism of resistance to dual VEGF–Ang2 blockade (11, 81). It further justifies the combination of antiangiotherapy and ICB. However, anti-VEGFR2 combined with anti-PD-L1 antibodies failed to improve survival in a glioblastoma model (259), and this lack of efficacy was attributed to a low incidence of high endothelial venules (263). Moreover, neither ICB nor anti-VEGF therapy was proved to be effective in highly desmoplastic tumors, such as cholangiocarcinoma and pancreatic ductal adenocarcinoma (20, 264). These different results suggest that vascular effects depend on tumor location and type, and more accurate measures of vascular normalization are required to determine the synergistic effect of immunotherapy and antiangiogenic therapy on tumor vascular normalization.

## Markers of Vascular Normalization

Given that there is no consensus on the types and the dosages of drugs used to normalize blood vessels, and because dose and duration of treatment may rely on the types and sizes of the tumors, vascular densities, expression levels of proangiogenic growth factors, and the conditions of the patients, it is critical to find and identify quantifiable methods to “normalization” (265, 266). With regard to antiangiogenic therapy, higher than the optimal dose of antiangiogenic drugs leads to excessive tumor vessel regression, further aggravating tumor hypoxia, while lower than the optimal dose cannot promote the normalization of tumor vessels (64, 267). Thus, a better understanding of the time window for vascular normalization will help determine appropriate drugs and dosages. Presented here are several imaging technologies or markers that can assess the window for vascular normalization. The morphological changes of blood vessels in tumor tissues can be observed by means of traditional histochemistry and the use of intravital microscopy to track the changes of vascular network over time, of which both are invasive and yield no information on vascular function, making it very difficult to apply them in clinical practice (265, 266). With the window of tumor vascular normalization dynamically monitored, the changes of tumor vascular perfusion can be detected by the noninvasive imaging technology that is also feasible in clinical practice. The perfusion and the window for blood vessel normalization were detected by using MRI (DCE-MRI and BOLD-MRI), dynamic contrast-enhanced ultrasonography (DCE-US), endomicroscopy, computed tomography, and positron emission tomography (PET), etc. (268–273), with the functionality of tumor blood vessels and the ideal regimen required to achieve normalization determined with the dynamic contrast-enhanced magnetic resonance imaging (DCE-MRI) and the specific radiotracer 18F-MISO developed for PET (13, 274, 275).

In addition to imaging techniques, serum-based biomarkers also provide a possibility for monitoring tumor vascular normalization windows. The serum level of soluble VEGFR (sFlt1), which is produced by ECs to finalize angiogenesis for maturation of neovasculature, was reported as a potential predictive marker to detect vascular normalization (199, 276). Ang1/Ang2 ratio is correlated with the degree of vascular normalization and may predict the degree of vascular maturation (66, 277). Apelin, an easily measured secreted protein whose expression is regulated by hypoxia, is overexpressed in many human cancers, including colon adenocarcinoma, nonsmall cell lung cancer, prostate cancer, and hepatocellular carcinoma. Apelin mRNA expression and plasma apelin levels were found in a preclinical study to reduce during the vessel normalization window induced by bevacizumab, indicating that apelin can be used as a potential indicator to identify the window of vascular normalization (210, 278–280). Despite the fact that the patients were reported to have higher levels of circulating type IV collagen in their blood when antiangiogenic therapy was efficient, and the intratumorale expression of P1GF was augmented during the gradual restoration of normalization of blood vessels (276, 281), all of the biomarkers proposed in recent years to measure the normalization of blood vessels have certain disadvantages, showing that, currently, there is no universally accepted method for identifying tumor window for vascular normalization. In **Table 2**, we list some imaging methods and serum markers that may monitor the normalization of tumor vessels and highlight their advantages and disadvantages.

**Table 2:** The pros and cons of some vascular normalization testing methods.

Methods		Pros	Cons	References
Imaging methods	Dynamic MRI	Observes perfusion and permeability; assesses tumor hypoxia; is extensively used	Is influenced by many factors, such as scanning schedule and movement	(270, 282, 283)
	Dynamic contrast-enhanced ultrasonography	Enables quantitative assessment of solid tumor perfusion; no radiation	Cannot clearly show the structure of blood vessels; poor sensitivity to changes in blood flow	(284–286)
	CT perfusion imaging	Measures the vascular structure and perfusion	Is susceptible to movement during data acquisition; radiation exposure; allergic reaction	(287–289)
	PET	Observes the vascular perfusion and permeability; assesses tumor hypoxia; reveals supplementary information on tumor growth and metabolism; sensitive	High rate of glucose metabolism in normal tissue and increased glucose uptake in inflammatory cells can affect image quality; nuclear radiation; expensive	(290, 291)
Serum markers	sFlt1	Inhibits VEGF activity	Is difficult to detect when the concentration in plasma is at a low level	(199, 276)
	Apelin	Upregulates when VEGF is overexpressed	Is influenced by body mass index and increases in obese patients	(265, 292)
	Ang	Compensates for the inhibition of the VEGF/VEGFR2 pathway	Production depends on the tumor heterogeneity, on the type of tumor, and on the type of antiangiogenic drug	(7, 293, 294)
	TSP-1	Increases in response to hypoxia, but decreases when hypoxia is alleviated	Interacts with multiple signaling receptors and with angiogenic and immune-modulatory factors in the extracellular matrix	(112, 295)
Intravital microscopy		Observes blood flow, blood vessel density, and permeability directly; can be used in combination with fluorescent probes, etc.	Cannot obtain fully quantifiable data on vascular function; requires microendoscopy to detect deep-seated tumors	(296, 297)
Histochemistry		Observes morphological changes in vascularity directly	Yields no information on vascular function; invasive	(265, 266)

VEGF, vascular endothelial growth factor.

## Conclusion

The U.S. Food and Drug Administration approved bevacizumab, the first angiogenesis inhibitor, to treat metastatic colorectal cancer. Targeting tumor vessels has aroused the interest of a growing number of researchers because of its great potential in tumor therapy. Although antiangiogenic therapy has increased progression-free survival of patients in many types of cancers, the overall results indicated that overall survival improvement was very limited and can be considered worse as long-term antiangiogenic therapy in cancer patients could lead to toxicity and drug resistance, and discontinuation of antiangiogenic drugs might lead to rebound effects that would further aggravate tumor invasion and metastasis. Targeting tumor vascular normalization can not only overcome the shortcomings of antiangiogenic therapy but also enhance the anticancer effect when combined with radiotherapy, chemotherapy, and immunotherapy.

One of the keys to maintaining tumor vascular normalization is to keep the balance between proangiogenic factors and antiangiogenic factors: for one thing, although inhibiting proangiogenic factors such as VEGF and Ang2 have been extensively studied to induce vascular normalization, there is, owing to their dose dependence, the lack of advanced technologies or biomarkers to determine the window for vascular normalization posing the major challenge; for another, angiostatic factors such as TNF, TSP-1, and endostar have shown the potential to promote vascular normalization. A particular example is TSP-1, whose expression can be regulated by nonpharmacological means of aerobic exercise, which is a good adjuvant therapy in combination with other methods in the process of promoting vascular normalization. Targeting miRNAs and endothelial cell metabolism provides a novel and exciting but challenging way to achieve vascular normalization, which has become a research hot spot in recent years. Aside from the methods of vascular normalization, the degrees of normalization are also associated with the types of tumors, the duration of treatments, and the types and dosages of the drugs. The current possible methods are summarized in detail for judging vascular normalization. For the purpose of enhancing the persistence of vascular normalization and overcoming the toxicity and drug resistance that may result from monotherapy, future research on vascular normalization might be focused more on the combination of different approaches, indicating that the key to normalizing tumor blood vessels is selecting an appropriate combination of these methods or with anticancer strategies (including surgery, chemotherapy, radiation therapy), as well as selecting reasonable order and time and appropriate drug dosage in the combination therapy. However, these therapies have complex biological effects, and their combinations may pose a risk of increasing toxic side effects to patients. Thus, it is necessary to find more evidence to confirm whether the same effect can be achieved after the transition to clinical application, despite the therapeutic benefits of tumor vascular normalization demonstrated in many preclinical studies.

In light of the importance of blood vessels to tumors, targeting tumor blood vessels is certain to play a crucial role in future tumor therapy. Hence, in this review, the methods of vascular normalization and the main challenges are described in detail, and the possible imaging techniques and biomarkers for evaluating vascular normalization are also introduced, which will provide references for further research directions of researchers.

## Author Contributions

TY developed the idea and drew the figures. All authors wrote the manuscript. HX and XG reviewed the manuscript and approved the final manuscript. All authors contributed to the article and approved the submitted version.

## Funding

This work was supported by grants to XG from The Special Supported Project of the Fourth Affiliated Hospital of Harbin Medical University [HYDSYTB201908].

## Conflict of Interest

The authors declare that the research was conducted in the absence of any commercial or financial relationships that could be construed as a potential conflict of interest.

## Publisher’s Note

All claims expressed in this article are solely those of the authors and do not necessarily represent those of their affiliated organizations, or those of the publisher, the editors and the reviewers. Any product that may be evaluated in this article, or claim that may be made by its manufacturer, is not guaranteed or endorsed by the publisher.
